# Intraperitoneal Perfusion with Cisplatin or Mitomycin C Improves Survival in Mice Bearing Peritoneal Metastases from Ovarian Cancer

**DOI:** 10.1245/s10434-025-18025-x

**Published:** 2025-08-11

**Authors:** Yvonne Andersson, Emil Løvstakken, Theodor Malmer Herud, Stein Waagene, Kjersti Flatmark, Karianne Giller Fleten

**Affiliations:** 1 Department of Tumor Biology, Institute of Cancer Research, The Norwegian Radium Hospital, Oslo University Hospital, Oslo, Norway; 2 Faculty of Medicine, Institute of Clinical Medicine, University of Oslo, Oslo, Norway; 3 Department of Surgical Oncology, The Norwegian Radium Hospital, Oslo University Hospital, Oslo, Norway

**Keywords:** HIPEC, Peritoneal metastases, Ovarian cancer, Hyperthermia, In vivo

## Abstract

**Background:**

Hyperthermic intraperitoneal chemotherapy (HIPEC) following cytoreductive surgery is a potentially curative treatment for patients with peritoneal metastases. Currently, there is no standardized protocol for performing HIPEC and there is large variation in the key parameters. In vivo models can be a valuable tool to better understand the impact of these parameters and how to improve this treatment strategy.

**Methods:**

Peritoneal tumors were established in immunodeficient mice by intraperitoneal injection of the high-grade serous human ovarian cancer cell line B76. Thirty-minute perfusion with cisplatin or mitomycin C at 37 or 41 °C was performed 4 days later. Treatment efficacy was assessed by measuring bioluminescence and overall survival.

**Results:**

We successfully established a closed peritoneal chemotherapy perfusion model in mice bearing peritoneal metastases. Perfusion with cisplatin and mitomycin C significantly inhibited tumor growth and increased overall survival by 38–48%. The addition of hyperthermia did not improve survival, although a clear synergistic effect with hyperthermia was observed in vitro for both drugs.

**Conclusion:**

The results suggest that intraperitoneal perfusion of cisplatin and mitomycin C can be a valuable adjuvant to cytoreductive surgery, while the addition of moderate hyperthermia did not improve efficacy. Additional studies investigating long-term outcome are necessary to determine the impact of hyperthermia as a component of the HIPEC procedure.

**Supplementary Information:**

The online version contains supplementary material available at 10.1245/s10434-025-18025-x.

The peritoneum is a common metastatic site for several abdominal cancers, including ovarian cancer, and peritoneal metastasis is associated with poor prognosis and poor response to systemic chemotherapy.^1^ A potentially curative treatment is cytoreductive surgery (CRS) followed by hyperthermic intraperitoneal chemotherapy (HIPEC), where CRS is performed to remove all visible tumors and HIPEC is administered to eliminate small residual tumors and free-floating tumor cells. Hyperthermia is thought to enhance the uptake and cytotoxic effect of drugs, inhibit DNA repair, and activate the immune system, but its efficacy as a component of HIPEC has not been proven.^2^

CRS-HIPEC has been shown to increase survival compared with CRS alone in studies in ovarian^3–5^ and colorectal cancer,^6,7^ but negative results have also been published.^8–10^ A potential confounding aspect is that there is no standardized protocol for performing HIPEC and there is large variation regarding key treatment parameters, such as choice of drug, drug concentration, duration of perfusion, choice of carrier solution, solution volume, and temperature.^11^ The impact of these parameters is unknown and in vivo models could potentially be a tool to better understand how to improve this treatment strategy.

In the current study, we established a functional closed perfusion model in mice bearing peritoneal metastases from the B76 ovarian cancer cell line, which accurately mimics many features of the procedure in patients. Furthermore, we determined tolerability of hyperthermia in the model and evaluated how adding moderate hyperthermia influenced survival and tumor growth after treatment with two drugs commonly used in HIPEC, cisplatin and mitomycin C (MMC).

## Materials and Methods

### Cell Line

The human ovarian cancer cell line B76 was a gift from Dr. C. Marth (Innsbruck Medical University, Innsbruck, Austria). B76 cells were transduced with a retroviral vector containing lmg* as the reporter gene. Lmg* is a dual reporter providing GFP and luciferase (kindly provided by Dr. Rainer Loew, EUFETS AG, Germany) as described previously.^12,13^ The cells were grown in RPMI-1640 supplemented with 10% fetal bovine serum and 2 mM L-glutamine (all from Sigma-Aldrich) in a humidified atmosphere at 37 °C with 5% CO_2_. Cells were routinely tested for mycoplasma and cell ID.

### In Vitro Studies

Cells were detached and suspensions (300,000 cells/mL) were mixed with medium containing increasing concentrations of cisplatin (Accord, London, UK) or MMC (Medac, Wedel, Germany) and incubated in water baths at 37 or 42 °C for 90 min. Cells were then sedimented by centrifugation at 1000 rpm for 5 min, washed once with medium and seeded in triplicate (15,000 cells/100µL) in 96-well plates. After 72 h, viability was assessed using MTS (Promega, Madison, WI, USA). IC_50_ was defined as the required drug concentration needed to reduce viability to 50% compared with untreated cells at 37 °C. Thermal enhancement ratios were calculated by dividing the IC_50_ value at 37 °C by the IC_50_ value at 42°C.^14^

### In Vivo Establishment of Peritoneal Metastases

B76 GFP/luciferase cells (2.5 mill in 500 µL) were injected intraperitoneally in mice. To mimic the state of the peritoneal cavity after CRS, i.e. with minimal intraperitoneal tumor burden, day 4 was chosen for treatment initiation, when peritoneal tumors were <2 mm.^15^ All procedures and experiments involving mice were approved by the Norwegian Food Safety Authority (application ID #28853) and were conducted according to the recommendations of the European Laboratory Animals Science Association and the ARRIVE guidelines.^16^ Female athymic nude foxn1^nu^ mice (6–8 weeks) were bred at the Department of Comparative Medicine, The Norwegian Radium Hospital, and kept in a specific pathogen-free environment at a constant temperature (22 ± 1 °C) and humidity (62 ± 5%), and with 15 air changes/hour and a 12 h light/dark cycle. A maximum of eight mice were housed in each cage. Food and water were supplied *ad libitum*, and mice were given cardboard houses and paper for environmental stimulation.

### Hyperthermic Intraperitoneal Chemotherapy (HIPEC) Setup

**Fig. 1:**
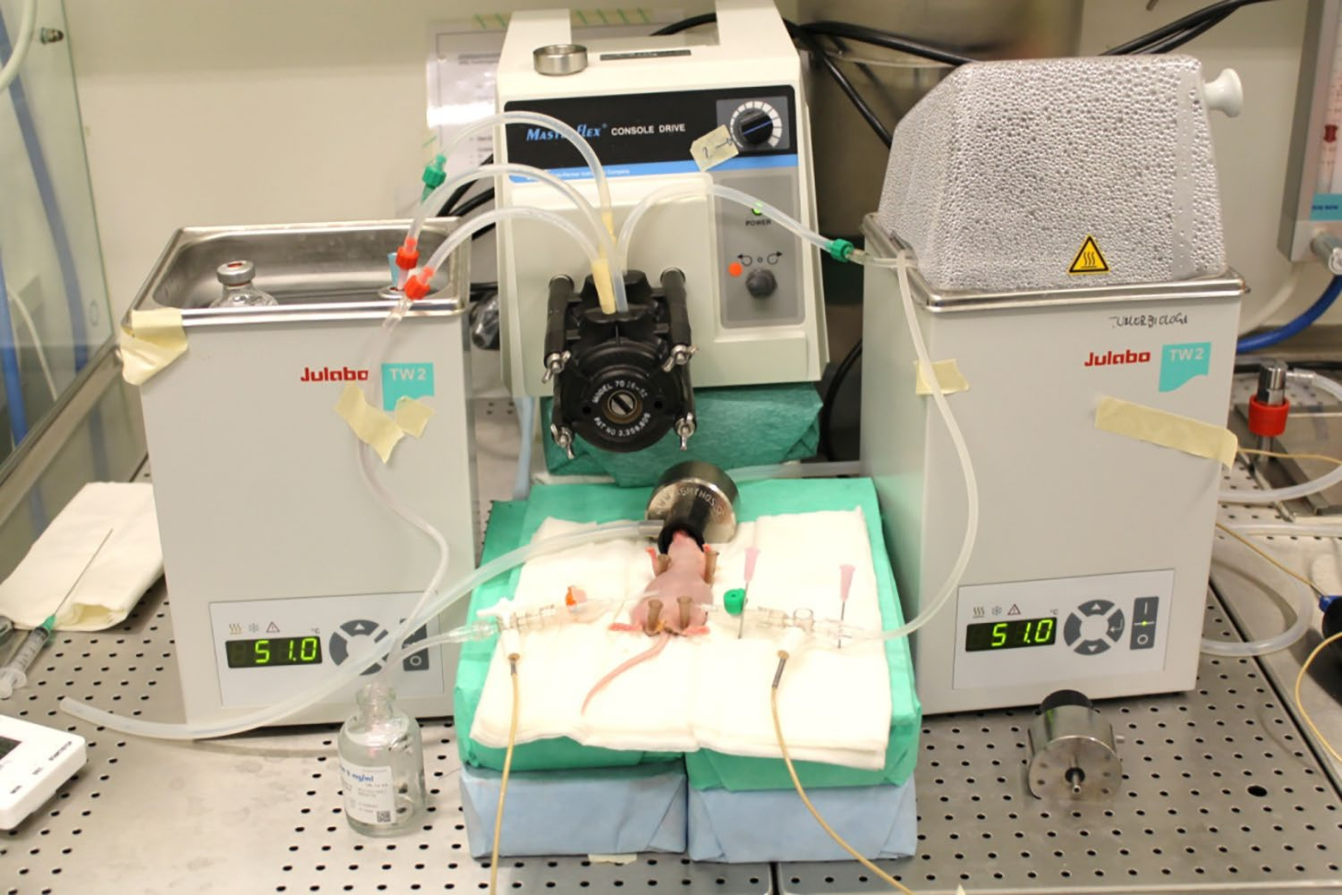
HIPEC setup. A glass vial containing saline or drug was heated in a water bath. The perfusate was circulated using a peristaltic pump into a second water bath where a standardized portion of the tube was immersed before entering the peritoneal cavity through the inflow catheter. From the peritoneal cavity, the perfusate was left to passively siphon into an empty glass vial, after which it was actively pumped back into the original vial in the water bath. Temperature probes were placed in the ports of the in- and outflow catheters. *HIPEC* hyperthermic intraperitoneal chemotherapy

A peritoneal perfusion circuit was established using silicon tubes connected to a glass vial holding the perfusion fluid, and to the peritoneal cavity with inflow and outflow catheters placed in opposite flanks. An 18G inflow catheter was inserted into the peritoneal cavity in the left lower abdominal quadrant, and a 14G outflow catheter was inserted between the upper and lower right abdominal quadrant, aiming to obtain as much distance between the two catheters as possible. Both catheters were connected to three-way ports, allowing insertion of temperature probes to measure in- and outflow temperatures. The vial with perfusion liquid and part of the silicon tubes were submerged in a water bath. A stable flow rate of 6 mL/min was achieved using a Masterflex console Drive peristaltic pump (Cole Parmer, Vernon Hills, IL, USA) (Fig. 1). The temperature in the water baths was kept at 51 °C for HIPEC and 47 °C for normothermic intraperitoneal chemotherapy (NIPEC) experiments, which was shown to provide stable intraperitoneal temperature conditions in subsequent experiments (see the Results section).

### HIPEC Procedure

Mice were anesthetized with 5% sevoflurane, and 1.5 mL 0.9% NaCl was injected intraperitoneally to allow safe insertion of the inflow catheter, after which the intraperitoneal volume was increased to 3 mL and the outflow catheter was inserted, establishing the perfusion circuit with saline. After a temperature stabilization period of 5 min, cisplatin or MMC was added to the perfusion fluid to achieve a final perfusate concentration of 40 µg/mL cisplatin or 20 µg/mL MMC and a total perfusate volume of 30 mL. The HIPEC procedure lasted for 30 min, with temperature readings at the in- and outflow catheters every 10 min. After the procedure was finished, perfusion with 0.9% NaCl only was performed for 3 min to remove the drug. Buprenorphine (0.15 mg/kg) was administered subcutaneously at the start of the HIPEC procedure for postoperative pain relief. Postoperatively, mice were weighed regularly and were sacrificed by cervical dislocation if displaying abdominal distension, weight loss >15%, or a body conditioning score of 2.^17^

### Luminescence Imaging

Luminescence images were obtained using the in vivo imaging system IVIS spectrum (Perkin Elmer, Waltham, MA, USA) to evaluate tumor growth during the experiments. Mice were injected with 200 μL luciferin (20 mg/mL; Biosynth, Staad, Switzerland diluted in phosphate-buffered saline) intraperitoneally 10 min before imaging. Both dorsal and ventral images were taken and the average of these images were used for analyses. Analyses of the images were performed using Living Image Software (Perkin Elmer).

### Statistical Analyses

Survival curves were generated using the Kaplan–Meier method and compared using the log-rank test. Differences in tumor weight were calculated using the two-tailed *t*-test, with Welch correction when necessary. *P*-values <0.05 were considered significant. Analyses were performed using GraphPad Prism v9 (GraphPad Software, LaJolla, CA, USA).

## Results

### In Vitro Treatment

**Fig. 2:**
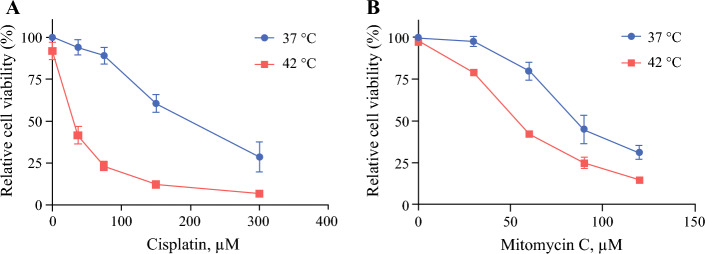
Cell viability of B76 cells measured 72 h after a 90 min exposure to increasing concentrations of **a** cisplatin and **b** mitomycin C at 37 or 42 °C. Error bars indicate the mean ± standard error of the mean (*n* = 3)

In vitro, hyperthermia (42 °C) alone did not reduce the viability of B76 cells (Figs. 2a, b) compared with normothermia (37°C). Both cisplatin and MMC effectively reduced cell viability, with IC_50_ values of 200 and 86 µM, respectively. For both cisplatin and MMC, the addition of hyperthermia further reduced cell viability, with IC_50_ values of 30 and 53 µM, respectively, corresponding to temperature enhancement ratios of 6.6 and 1.6, respectively (Fig. 2).

### Optimization of the HIPEC Procedure

Several measures were taken to optimize the HIPEC procedure, with the main challenge being to avoid obstruction of the outflow catheter. One improvement was to make four small perforations in the catheter to help the liquid run through. Another improvement was to introduce passive outflow, where the perfusate was siphoned from the peritoneal cavity into an empty vial before it was re-introduced into the perfusion circuit. These adjustments completely prevented obstruction of the outflow catheter, a problem several researchers have encountered when performing HIPEC in mice.^18,19^

**Fig. 3:**
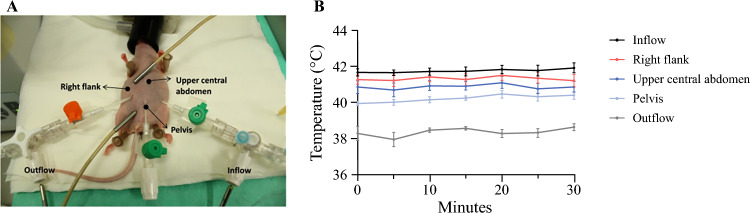
Results from optimization experiments to evaluate intraperitoneal temperature during HIPEC. **a** Image showing the placement of the intraperitoneal temperature probes. **b** Temperature at three different locations in the peritoneal cavity and in the in- and outflow. Temperatures were measured every 5 min. Error bars represent the mean ± standard error of the mean (*n* = 6). *HIPEC* hyperthermic intraperitoneal chemotherapy

Normothermic perfusion was extremely well tolerated, while the addition of hyperthermia led to short-term morbidity and mortality in initial experiments. Continuous intra-abdominal temperature measurement during the actual HIPEC procedures was impractical, adding unacceptable risk of inadvertent damage to organs to an already complex procedure. To establish safe and stable hyperthermic conditions, a separate set of experiments was setup to define the exact temperature that would be tolerable for the animals. After establishing a stable perfusion circuit, intra-abdominal temperature was monitored at three separate locations in addition to the in- and outflow catheters (Fig. 3a). The intraperitoneal temperature was adjusted by changing the temperature in the water baths, while keeping all other parameters stable (perfusion volume, tube length, and catheter placement). The maximum tolerated water bath temperature that was compatible with animal short-term survival with acceptable morbidity was 51 °C. With this setting, the mean intraperitoneal temperatures were between 40.2 and 41.3 °C, with corresponding temperatures of 41.7 and 38.3 °C measured at the inflow and outflow catheters, respectively (Fig. 3b). A water bath temperature of 51 °C was therefore chosen for all subsequent HIPEC experiments, and in- and outflow temperatures were monitored in each experiment as a means of experimental control. The in- and outflow temperatures were similar in treatment experiments. In the experiments with cisplatin, the mean in- and outflow temperatures were 41.7 °C and 38.0 °C, respectively, while in experiments with MMC, the temperatures were 41.7 and 38.7 °C, respectively. A water bath temperature of 47 °C was chosen for all NIPEC experiments, to have a 4°C temperature difference between NIPEC and HIPEC treatments. In the experiments with cisplatin, the mean in- and outflow temperatures were 37.6 and 34.7 °C, respectively, while in experiments with MMC, the temperatures were 37.4 and 35.1 °C, respectively.

### In Vivo Perfusion with Cisplatin

**Fig. 4:**
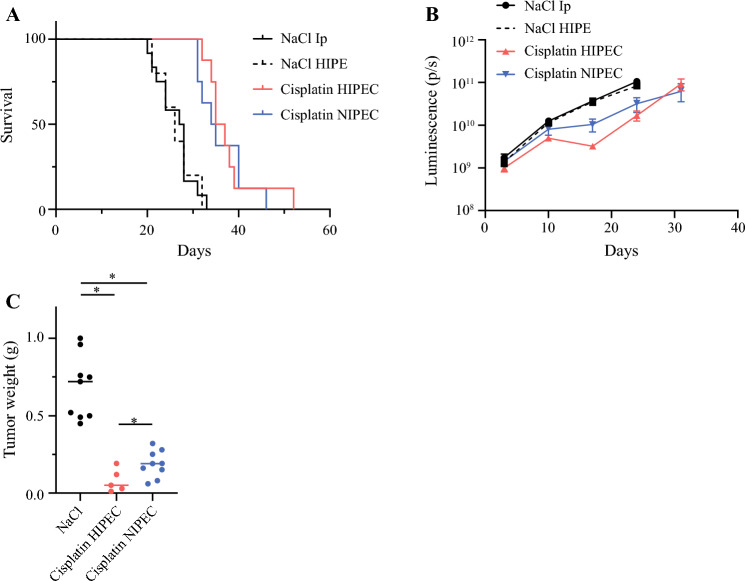
**a** Kaplan–Meier survival curve comparing mice treated with NaCl intraperitoneally, NaCl HIPE, cisplatin HIPEC, or cisplatin NIPEC. **b** Quantification of total flux luminescence (p/s) in B76 xenografts treated with NaCl intraperitoneally, NaCl HIPE, cisplatin HIPEC, or cisplatin NIPEC. Error bars indicate the mean ± standard error of the mean (*n* = 5–12). **c** Tumor weight in mice treated with NaCl, HIPEC cisplatin, or NIPEC cisplatin. Median is indicated (*n* = 5–9). * indicates *p* < 0.05. *HIPEC* hyperthermic intraperitoneal chemotherapy, *NIPEC* normothermic intraperitoneal chemotherapy, *p/s* photons/second

Mimicking HIPEC conditions with saline for 30 min did not influence overall survival in mice compared with a single intraperitoneal injection of saline (median survival 26 days vs. 27.5 days; *p* = 0.87), and single intraperitoneal injection of saline was used as a control in subsequent experiments. Perfusion of the peritoneal cavity with cisplatin 40 µg/mL significantly increased the survival compared with the control to 36 and 34.5 days for HIPEC and NIPEC, respectively (Fig. 4a), but adding hyperthermia did not significantly improve survival. Interestingly, when intraperitoneal tumor growth was monitored by luminescence measurement, a slight reduction of signal was detected in the HIPEC group between days 15 and 20 (Fig. 4b). To investigate this observation further, another experiment was conducted where animals were sacrificed on day 17. In this experiment, the mean tumor weight was slightly lower in the HIPEC group compared with the NIPEC group (0.08 g vs. 0.19 g, respectively; *p* = 0.039), and also compared with the control (0.68 g; *p* < 0.001) [Fig. 4c].

### In Vivo Perfusion with Mitomycin C

**Fig. 5:**
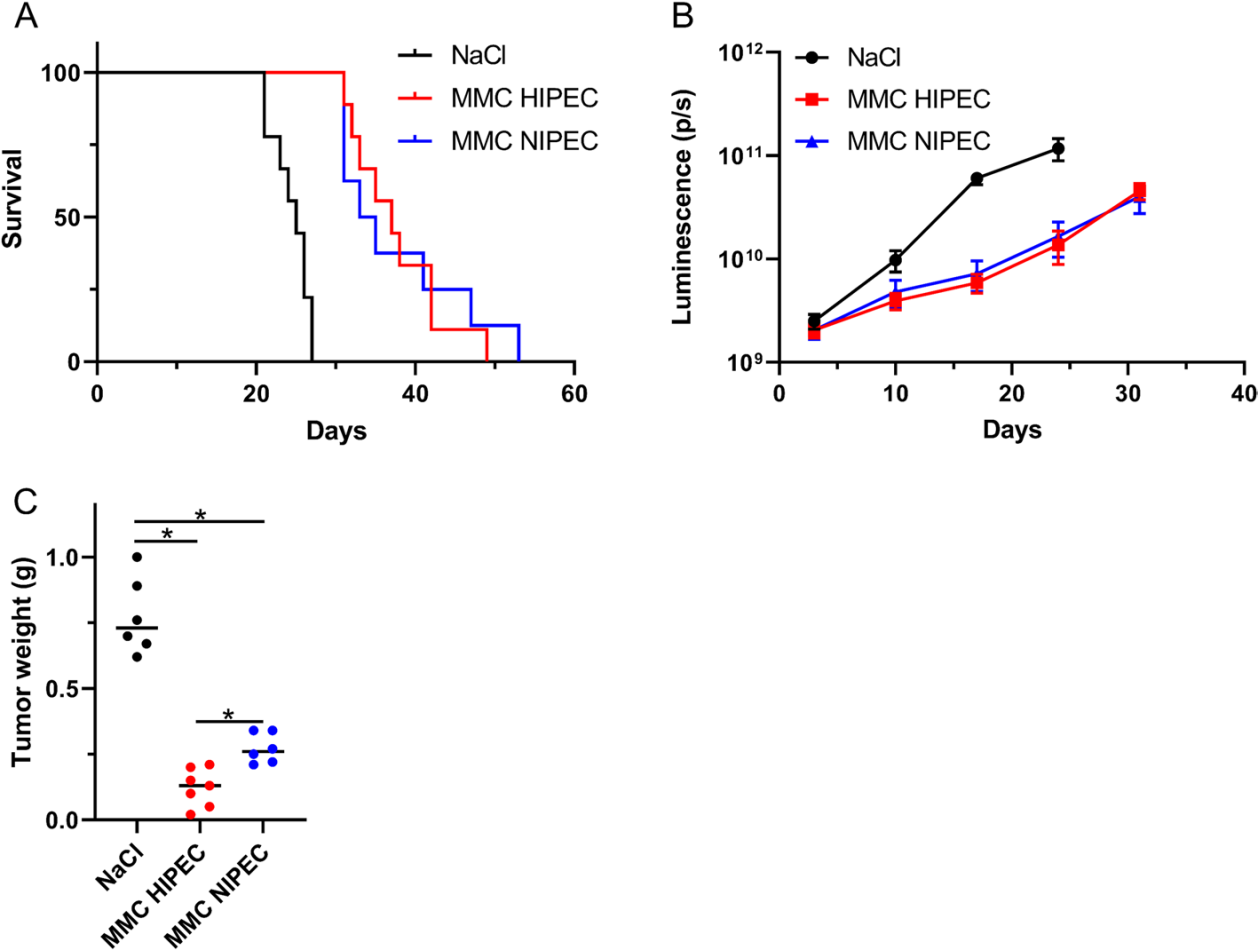
**a** Kaplan–Meier survival curve comparing mice treated with *NaCl* intraperitoneally, *MMC HIPEC*, or *MMC NIPEC*. **b** Quantification of total flux luminescence (p/s) in B76 xenografts treated with *NaCl* intraperitoneally, *MMC HIPEC*, or *MMC NIPEC*. Error bars indicate the mean ± standard error of the mean (*n* = 8–9). **c** Tumor weight in mice treated with *NaCl*, *MMC HIPEC*, or MMC NIPEC. Median is indicated (*n* = 6–7). * indicates *p* < 0.05. *MMC* mitomycin C, *HIPEC* hyperthermic intraperitoneal chemotherapy, *NIPEC* normothermic intraperitoneal chemotherapy, *p/s* photons/second

Perfusion of the peritoneal cavity with MMC 20 µg/mL (HIPEC or NIPEC) significantly increased survival compared with the control (25 days) to 37 and 34 days, respectively (Fig. 5a). Adding hyperthermia did not significantly increase survival (*p* = 0.93). Intraperitoneal tumor growth was again monitored by luminescence measurement. A decrease in signal was detected between the control and HIPEC/NIPEC, but there was no difference between HIPEC- and NIPEC-treated mice (Fig. 5b). Due to the observed differences in tumor weight at day 17 with cisplatin, the same experiment was performed with MMC. The mean tumor weight was again slightly lower in the HIPEC group compared with the NIPEC group on day 17 (0.12 g vs. 0.28 g; *p* = 0.002) and also compared with the control (0.77 g; *p* < 0.0001) (Fig. 5c).

## Discussion

In this work, we successfully established an experimental model where a 30-min intraperitoneal perfusion with cisplatin and MMC was administered with or without the addition of moderate hyperthermia to mice bearing peritoneal metastases from human ovarian cancer. The main findings were a strong growth inhibitory effect of intraperitoneal perfusion with improved survival of the animals, while the addition of hyperthermia did not improve survival, although both drugs exhibited synergy with hyperthermia in vitro.

Intraperitoneal perfusion with both cisplatin and MMC increased the survival of mice substantially, with similar extension of animal survival of 38–48%, showing that both drugs effectively inhibited tumor growth. After the PRODIGE7 trial failed to show benefit of 30 min oxaliplatin-based HIPEC in oxaliplatin pretreated patients with colorectal cancer, HIPEC has become a controversial procedure, with some centers abandoning its use.^8,20^ However, the results from the PRODIGE7 trial may not be valid for other drugs, treatment settings, and cancer types,^21^ and it may therefore be premature to dismiss all potential HIPEC regimens based on one negative trial. Other clinical trials, particularly in ovarian cancer, which are in line with results from our study and other in vivo experiments, strongly suggest that intraperitoneal perfusion of other cytotoxic drugs, such as cisplatin and MMC, could still be a valuable addition to CRS.^3,22–25^

The addition of hyperthermia did not improve survival for either of the two investigated drugs in this model. The slight growth inhibition observed at day 17 is difficult to interpret, but it would indicate that the experimental design in such studies may be vital, i.e. choosing an early endpoint might result in transient effects that will not translate into a true survival benefit. Early endpoints are chosen in a large proportion of published studies investigating HIPEC in murine models, particularly when the focus is on understanding the molecular mechanisms of hyperthermia,^26–29^ while some studies have included survival endpoints. In a gastric cancer model, 50-min HIPEC at 40 °C combining cisplatin and MMC improved survival compared with normothermic perfusion.^30^ Interestingly, in an immunocompetent model of colorectal cancer, a 15 min perfusion with nanoparticles containing a heat shock protein 90 (HSP90) inhibitor at 41–43 °C resulted in markedly improved survival compared with normothermic perfusion.^19^ These results emphasize the promising concept of nanoparticle encapsulation for intraperitoneal drug delivery, and also the potential for application of other, non-cytotoxic drugs in this setting. Varying results of adding hyperthermia have also been observed in studies performed in rats. In a patient-derived xenograft model of pseudomyxoma peritonei, 90 min HIPEC using MMC at 41 °C resulted in slightly increased overall survival compared with normothermia-treated animals.^31^ In contrast, the addition of hyperthermia did not improve survival in a study of colorectal cancer, while also using 90 min perfusion with MMC at 41 °C.^32^ Additional studies focusing on the long-term effects of hyperthermia are necessary to perform, and not only investigate the short-term benefits of the treatment.

In vitro, the cell viability of B76 cells was convincingly reduced by the addition of hyperthermia for both drugs, with the strongest temperature enhancement ratio being observed for cisplatin. The conflicting results between in vitro and in vivo exposure to hyperthermia could be related to the experimental setup. Without the restrictions caused by the tolerance levels in mice, it was possible to increase the in vitro temperature from 40.2–41.3 to 42 °C and increase the exposure duration from 30 to 90 min without negatively affecting the viability of cells exposed to hyperthermia only. Previous in vitro studies have shown a clear correlation between increased temperature and duration with improved sensitivity to drugs,^33^ and it is possible that such synergy could be achieved with higher temperatures. When the long-term effects are investigated, it is crucial that the mice tolerate the procedure, and a lower temperature may be necessary to avoid thermal damage to organs. With the challenges associated with acquiring sufficiently high temperatures and long enough exposure duration in mice, it is possible that mouse models are not the most suitable for investigating the addition of hyperthermia to intraperitoneal perfusion, at least if investigation of survival outcomes is the target.

The intraperitoneal temperature is a key parameter in HIPEC. The highest tolerable intraperitoneal temperature we were able to achieve was around 41 °C. When higher temperatures were used, some of the mice would suffer heat-related damage and had to be sacrificed 1–5 days after the procedure. The existing literature describing HIPEC experiments in mice was found to be highly divergent, with a plethora of experimental conditions being investigated. In particular, information regarding the intraperitoneal temperature was insufficiently described in several studies (summarized in electronic supplementary material Table 1). The reported HIPEC temperatures varied from 39 to 43°C, but there was also a large variation in reported locations for temperature measurement. Some studies measured intraperitoneal temperature, but without specifying the temperature probe location relative to entry of the perfusion liquid.^25,27,28,34–36^ As we have shown in this study, the measured intraperitoneal temperature will vary depending on placement of the probe relative to the entry of perfusate. Some investigators have measured the temperature in the inflow catheter,^26,37^ which, as we have shown, is likely to be higher than the actual intraperitoneal temperature. Others have reported the temperature of the water bath.^22–24,38^ As the temperature of the perfusion liquid will decrease while passing through the tubes to the peritoneal cavity, this is also not an accurate representation of the intraperitoneal temperature. Temperature heterogeneity in the peritoneal cavity is also a potential confounder with respect to the actual HIPEC temperature, and a high flow rate will facilitate temperature homogeneity.^39^ We were able to achieve a consistent flow rate of 6 mL/min, resulting in only moderate temperature differences within the peritoneal cavity, while previous studies have reported flow rates as low as 1 mL/min. Taken together, existing experimental literature seems to exhibit similar procedural and registration variability with respect to reporting HIPEC temperatures, as we see in the clinical literature, where there are large variations in key parameters, including temperature, which can vary between 40 and 44 °C in different centers.^11^ In the OVHIPEC trial, where improved survival after CRS-HIPEC compared with CRS was shown, an intraperitoneal temperature of 40–42 °C was reported.^3,40^ This is similar to the temperature we achieved in our in vivo experiments without seeing a benefit of hyperthermia. A higher perfusion temperature of 42°C is used in the ongoing HyNOVA trial comparing perfusion with cisplatin administered as NIPEC (37 °C) or HIPEC. Progression-free and overall survival are among the endpoints, and this study will give further insight into the significance of hyperthermia in HIPEC.^41^ At present, there are still insufficient data to conclude whether hyperthermia at the level that is administered to patients in HIPEC increases the efficacy of intraperitoneal perfusion of chemotherapy in ovarian cancer.

## Conclusion

Our results show that intraperitoneal perfusion of chemotherapy can be a valuable addition to CRS, but we did not observe a survival benefit of adding moderate hyperthermia. The optimal temperature for hyperthermia, or if it improves treatment outcome, is still not determined, but it might be higher than what is tolerated in mice. Rats might therefore be a more suitable model for such experiments. Taken together with previous studies, our study highlights the need for additional research focusing on the long-term effects of hyperthermia, both in the preclinical and clinical setting, to determine if hyperthermia improves the treatment efficacy.

## Supplementary Information

Below is the link to the electronic supplementary material.

Supplementary file1 (DOCX 44 kb)
