# Supplementary material for: Intraperitoneal Perfusion with Cisplatin or Mitomycin C Improves Survival in Mice Bearing Peritoneal Metastases from Ovarian Cancer
Source: Ann Surg Oncol. 2025 Aug 11;32(12):9333–40. doi: 10.1245/s10434-025-18025-x (PMC12534361; doi:10.1245/s10434-025-18025-x)
Supplement: Supplementary file 1 — Supplementary file1 (DOCX 44 kb) [file 10434_2025_18025_MOESM1_ESM.docx]

Supplementary Table 1. Overview over drug, control groups, mouse strain, cell line, reported temperature, location of temperature measurement, flow rate, endpoint and results in HIPEC experiments. Only experiments using an open or closed technique with circulation of perfusate are included.

| **Reference** | **Drug** | **Control groups** | **Mouse strain/ cell line** | **Reported temperature** | **Location of measured temperature** | **Duration (min)** | **Flow rate** | **Open/closed** | **Endpoint** | **Results** |
| --- | --- | --- | --- | --- | --- | --- | --- | --- | --- | --- |
| **Studies reporting on overall survival with HIPEC compared to NIPEC treatment.** | | | | | | | | | | |
| Wang et. al.[19] | HSP-90 nanoinhibitor | Perfusion HT, NIPEC | BALBc/ CT26-luc | 41-43°C | Inflow+I.p | 15 | 5 mL/min | Closed | OS + immune stimulation day 17 | **OS**:HIPEC > NIPEC |
| Graziosi et. al. [30] | Cisplatin, MMC | Untreated, NIPEC | NOD-SCID/ MKN45 | 40°C | Not specified | 50 | 4 mL/min | Open | OS + Gene expression day 10 | **OS:**HIPEC > NIPEC |
| **Studies reporting on overall survival.** | | | | | | | | | | |
| Derrien et. al. [34] | Cisplatin | Untreated | NMRI nu/ SHIN -3-luc | 39°C | I.p | 60 | 3 mL/min | Open | OS | **OS:**HIPEC = control |
| Nevo et. al. [23, 24] | MMC, Thymosin α1 | Sham- operated, midline laparotomy | C57BL6 /MC38 | 43°C | Water bath | 20 | 1 mL/min | Open | OS + immune response day 14 | **OS:**HIPEC > control  **Immune infiltration:** HIPEC > control. |
| Geva et. al. [22] | MMC, PD-1 inhibitor | Sham-operated, midline laparotomy | C57BL6/ MC38 | 43°C | Water bath | 20 | 1 mL/min | Open | OS + immune response on day 5,7,10,12,14,20,23 | **OS:**HIPEC > control  **CD8:** HIPEC > control |
|  |  |  |  |  |  |  |  |  |  |  |
| Liesenfeld et. al. [35] | Oxaliplatin | Untreated | NCr Athymic/ HCT116, HT29 | 41-43°C | I.p | 30 | 5 mL/min | Closed | OS + apoptosis 24h | **OS:**HIPEC = control  **Apoptosis:** HIPEC = control |
| Lehmann et. al. [25] | Mitomycin C/doxorubicin, diethyldithiocarbamate | Saline perfusion 37°C | C57BL6 /MC38 | 40°C | I.p | 60 | - | Open | OS | **OS**:HIPEC > control |
| **Studies reporting on tumor weight** | | | | | | | | | | |
| Kudo et.al. [36] | Cisplatin | Saline perfusion 37°C | BALBc/ Colon26 | 43°C | I.p | 10-30 | 2.5 mL/min | Closed | Tumor weight day 10 | **Tumor weight:** HIPEC < NIPEC |
| Manoğlu et. al.[26] | MMC, 5-FU | Saline perfusion 37°C NIPEC, | NCr Athymic/ CC531 | 40.5±0.5°C | inflow | 45 | - | Closed | Tumor weight, + apoptosis day 5 | **Tumor weight:** HIPEC < NIPEC **Apoptosis:** HIPEC > NIPEC |
| Qui et. al. [27] | Raltitrexed | Saline perfusion 37°C, NIPEC | Nude/ HCT116, LOVO | 43°C | I.p | 30 | - | Closed | Tumor weight + apoptosis day 10 | **Tumor weight:** HIPEC < NIPEC **Apoptosis:** HIPEC> NIPEC |
| Gong et. al. [28] | Recombinant mutant human TNF-α, Raltitrexed | Saline perfusion 37°C, NIPEC | BALBc nude/ HCT116-luc | 42°C | I.p | 30 | - | Closed | Tumor weight + PCI, apoptosis day 28 | **Tumor weight:** HIPEC < NIPEC |
| **Studies reporting on drug distribution and apoptosis** | | | | | | | | | | |
| Muenyi et. al. [37] | Cisplatin | Untreated, NIPEC | NCr Athymic / A2780/CP70 | 43°C | Inflow | 60 | 3 mL/min | Closed | Drug distribution + protein expression 0 and 24 h | **Platinum uptake**: HIPEC =NIPEC |
| Carlier et. al. [29] | Cisplatin | Saline perfusion 42°C, NIPEC | NCr Athymic/ SKOV3 | 40–41°C | Unknown | 60 | - | Open | Drug distribution 0 h | **Platinum uptake:** HIPEC> NIPEC |
| Wagner et. al. [38] | Cisplatin | Saline perfusion 37 and 42°C , NIPEC | NOD/LtSz-scid IL2Rnull / RH-30 | 42°C | Water bath | 60 | 3 mL/min | Closed | Apoptosis 0 h | **Cl. casp3:** HIPEC=  NIPEC  **Ki67:** HIPEC=  NIPEC |

MMC-mitomycin C, HIPEC-hyperthermic intraperitoneal chemotherapy, NIPEC-normothermic intraperitoneal chemotherapy, OS-overall survival, PCI- peritoneal cancer index
